# Antiaging, Wound‐Healing Properties and Chemical Characterization of Crude Hydroalcoholic Extract and Fractions of *Myrcia neoobscura*

**DOI:** 10.1002/cbdv.202501417

**Published:** 2025-07-10

**Authors:** Larissa Mascarenhas Krepsky, Ana Helena Loos Moritz, Mayra Alice Corrêa Pitz, Letícia Bachmann, Diogo Alexandre Siebert, Paula Flegar, Stjepan Lešnjak, Ana Tirić, Amanda Tavares Germano, Mayara da Silva, Carlos Rafael Vaz, Larissa Benvenutti, André Luís de Gasper, José Roberto Santin, Isabel Daufenback Machado, Luciano Vitali, Marijana Zovko Končić, Michele Debiasi Alberton

**Affiliations:** ^1^ Fundação Universidade Regional de Blumenau Blumenau Santa Catarina Brazil; ^2^ Department of Pharmaceutical Sciences Fundação Universidade Regional de Blumenau Blumenau Santa Catarina Brazil; ^3^ Department of Pharmacognosy Faculty of Pharmacy and Biochemistry, University of Zagreb Zagreb Croatia; ^4^ Universidade do Vale do Itajaí Itajaí Santa Catarina Brazil; ^5^ Department of Chemistry Universidade Federal de Santa Catarina Florianópolis Santa Catarina Brazil

**Keywords:** collagenase, elastase, *Myrcia neoobscura*, polyphenols, wound healing

## Abstract

The aim of this study was to characterize the phytochemical profile and potential antiaging and wound‐healing activities of 70% hydroalcoholic crude extract (CHE) from *Myrcia neoobscura* leaves and its fractions—insoluble (IF), ethyl acetate (EAF), and aqueous (AF)—for use in phytocosmetics for skin application. CHE and its fractions showed high concentrations of total phenolics, including flavanols and flavonoids. Ten phenolic compounds were identified, with gallic acid as the major, followed by chlorogenic and *p*‐coumaric acids. Catechin, isoorientin, taxifolin, resveratrol, naringenin, and eriodictyol were reported for the first time in the genus. CHE and AF showed the best results in antioxidant assays. In antiglycation activity, EAF showed the highest oxidative glycation inhibition, comparable to quercetin. CHE and AF exhibited the strongest collagenase and elastase inhibition. CHE, IF, and EAF showed the highest SPF. CHE promoted 81.86% fibroblast migration in 24 h, indicating strong wound‐healing potential. CHE maintained cellular viability in the MTT assay. In the HET‐CAM assay and agarose overlay assays, CHE and fractions were classified as non to mildly irritating. These results suggest potential antiaging applications, mainly attributed to the phenolic profile.

## Graphical abstract

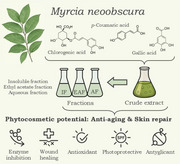

## 1 Introduction

The use of natural resources to treat skin diseases is an ancient practice, where the use of plants against diseases and for cosmeceutical purposes is used in popular medicine in many cultures [1, 2]. Care for skin health and aesthetic appearance is growing in modern society and makes the use of cosmetics part of daily routine [3].

The skin as it covers the body, is under the influence of the environment, such as the action of UVB and UVA rays, smoking, alcoholism, among other factors. Cutaneous aging can occur due to intrinsic factors, which occur naturally, as well as extrinsic factors, which are environmental factors to which all are exposed. Sagging, wrinkling, and loss of elasticity are characteristics of chronological aging [4]. Among the features of extrinsic aging are cutaneous hyperpigmentation leading to the photoaging, melasma, senile spots, melanomas and freckles [5]. There is a growing interest in the use of herbal medicines for antiaging uses making the search for new cosmetic actives of natural origin an alternative for skin care [6]. The products of plant secondary metabolism have attracted economic interest due to their chemical diversity and biological activities. Bioactive compounds have the potential to protect the epidermal and dermal layers of the skin, which are mainly composed of elastin and collagen, and are used in formulations for UV radiation protection, photoprotection, antioxidant, skin‐lightening effect, among others. Such bioactivities, have been shown to be related to polyphenolic compounds [2, 7].

Myrtaceae family is considered the ninth‐largest family of angiosperms, with diversity centers in humid tropics, primarily in South America [8]. It represents the largest family in the Brazilian flora, with 1200 species spread throughout the country [9]. The plants of this family are rich in phenolic compounds [10], that have potential to protect the epidermal and dermal layers of the skin and are utilized in UV protective formulas [2, 7]. Phenolics also possess antioxidant potential, whose strength is related to the number and position of hydroxyl groups present in their structures, as well as anti‐tyrosinase, anti‐elastase, and anti‐collagenase activity [11, 12].

The genus *Myrcia*, one of the largest within Myrtaceae, is a significant source of nonvolatile compounds such as triterpenes and phenolic compounds. Studies with *Myrcia* species indicate that they exhibit antioxidant activity primarily related to the presence of phenolic compounds and flavonoids. In addition, the extracts of *Myrcia* species displayed inhibition of melanogenesis and photoprotective properties, making them whitening and antiaging agents [8, 13, 14]. *Myrcia neoobscura* (E.Lucas & C.E.Wilson) is a native endemic tree in Brazil and lacks scientific studies. This study aims the phytochemical composition of the crude extract (CHE) and fractions of *M. neoobscura*, as well as to evaluate their potential for use as an active ingredient in phytocosmetic applications for skin care.

## 2 Results and Discussion

### 2.1 Quantitative Analysis of Secondary Metabolites of CHE and Fractions of *M. neoobscura* Leaves

For phytochemical characterization of the extract and the fractions of *M. neoobscura* leaves, the quantities of total phenolic compounds (TPCs), flavonoids compounds (TFC), and flavanols (TFLC) were assessed (Table 1). In this initial phytochemical characterization study of *M. neoobscura* leaves, it can be observed that aqueous fraction (AF) exhibited the highest levels of flavanols and TPCs. Ethyl acetate fraction (EAF) obtained the second highest phenolic contents and the highest flavonoid content. An interesting point to highlight is the result of the quantity of phenolic compounds found in insoluble fraction (IF). In this fraction, less polar compounds, insoluble in water, may be present. However, during the fractionation process, the precipitation of polyphenolic compounds, like tannins with higher molecular weight may have occurred. The results obtained from the phytochemical characterization are comparable to those found in the work of Dos Santos et al. [15] for other *Myrcia* species, where they quantified the content of TPC and TFC in hydroethanolic extracts of *Myrcia bella*, *Myrcia fallax*, and *Myrcia guianensis*. The TPC values of these species were 215.39 ± 0.01, 218.19 ± 0.81, and 71.54 ± 0.01 mgGA g^−1^, respectively; while TFC values were 20.24 ± 0.48, 25.92 ± 0.50, and 19.17 ± 0.30 mgQUE g^−1^, respectively.

**TABLE 1:** Phytochemical characterization of CHE and fractions of *Myrcia neoobscura* leaves.

Sample	TPC (mgGA g^−1^)	TFC (mgQUE g^−1^)	TFLC (mgCAT g^−1^)
CHE	169.5 ± 10.72^a^	2.86 ± 0.1.66^a^	71.53 ± 1.15^a^
IF	177.2 ± 15.20^a^	11.90 ± 2.00^a^	87.53 ± 9.45^a^
HF	25.02 ± 3.29^b^	ND	ND
DF	108.3 ± 19.90^c^	ND	14.2 ± 11.31^b^
EAF	233.5 ± 18.46^d^	39.68 ± 9.61^b^	8.2 ± 2.82^b^
AF	297.0 ± 11.0^e^	8.58 ± 1.56^a^	302.9±24.85^c^

*Note*: The results for TPC, TFC, TFLC are expressed as media ± SD of three independent experiments. Statistical analysis was performed using one‐way ANOVA followed by Tukey's test, results with equal letters did not differ statistically *p* ≤ 0.05.

Abbreviations: ND, non‐detected; TFC, total flavonoid content expressed as mg of quercetin per g of dried extract or fraction (mgQUE g^−1^); TFLC, total flavanol content expressed as mg of catechin per g of dried extract or fraction (mgCAT g^−1^); TPC, total phenolic content expressed as mg of gallic acid per g of dried extract or fraction (mgAG g^−1^).

**FIGURE 1:**
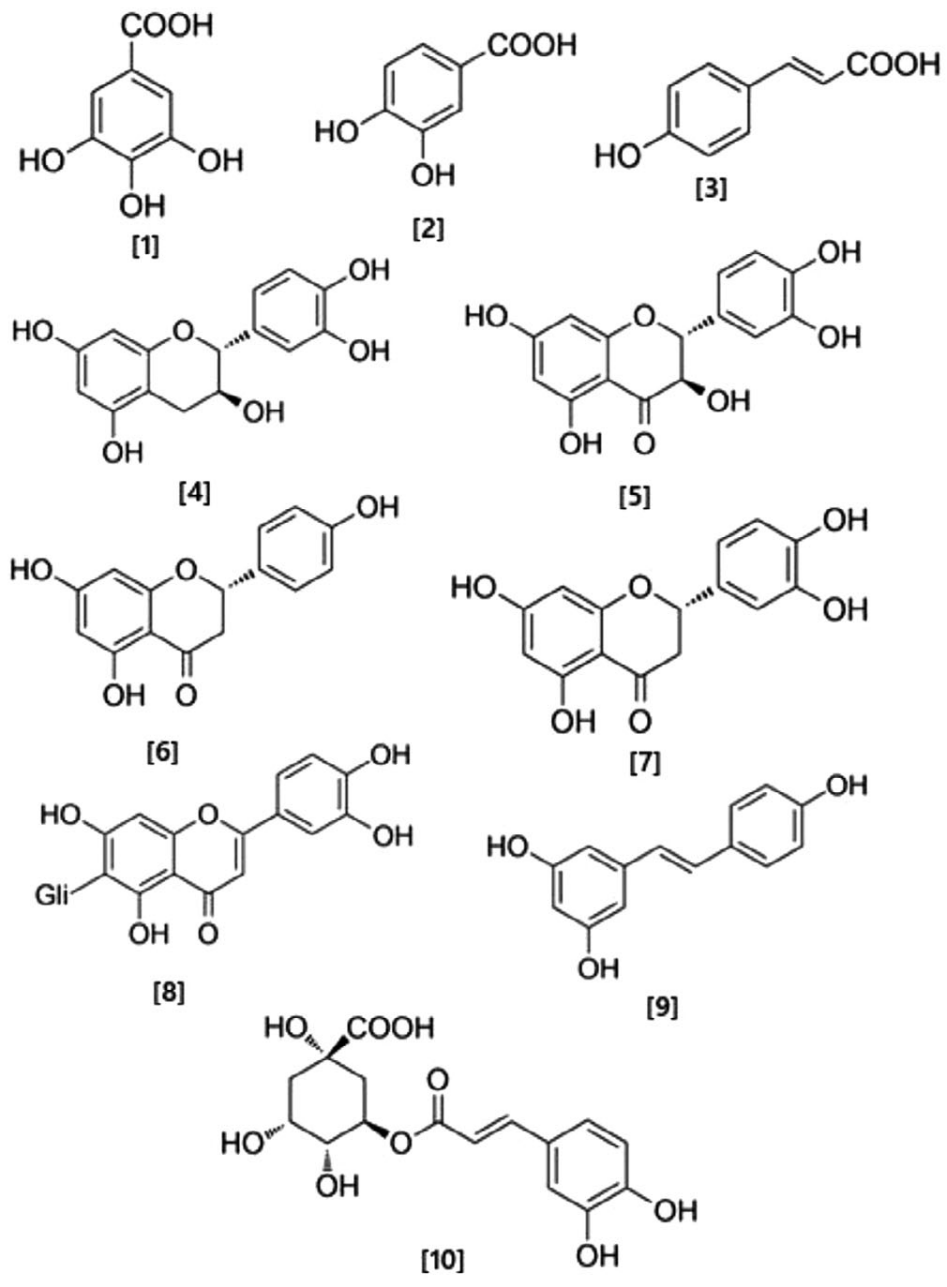
Identified phenolic in CHE of *Myrcia neoobscura* leaves. (1) Gallic acid; (2) protocatechuic acid; (3) *p*‐coumaric acid; (4) catechin; (5) taxifolin; (6) naringenin; (7) eriodictyol; (8) isoorientin; (9) resveratrol; (10) chlorogenic acid.

The results of the HPLC–ESI–MS/MS analysis of the CHE are presented in Table 2. Out of the 39 phenolic compounds analyzed, 10 were identified (Figure 1), with gallic acid, chlorogenic acid, and *p*‐coumaric acid being the main phenolic compounds present in the CHE of *M. neoobscura*.

**TABLE 2:** Identified phenolic (µg g^−1^) in CHE of *Myrcia neoobscura* leaves.

Phenolic compound	Rt (min)	MF	TM (Da)	EM [M−H]	MS/MS	Concentration (µg g^−1^)	Previous studies in *Myrcia*
Gallic acid	3.29	C_7_H_6_O_5_	170.12	168.9	125.0	42.6 ± 0.4	[16]
Protocatechuic acid	5.06	C_7_H_6_O_4_	154.12	152.92	109.00	> LOQ	[17]
Catechin	8.23	C_15_H_14_O_6_	290.26	289.0	109.0	> LOQ	New in genus
Chlorogenic acid	8.94	C_16_H_18_O_9_	354.31	353.1	191.0	5.4 ± 0.8	New in genus
Isoorientin	10.00	C_21_H_20_O_11_	448.38	446.8	326.9	> LOQ	New in genus
*p*‐Coumaric acid	10.02	C_9_H_8_O_3_	164.04	162.9	119.1	0.24 ± 0.2	[18]
Taxifolin	10.31	C_15_H_12_O_7_	304.25	303.0	125.1	> LOQ	New in genus
Resveratrol	10.87	C_14_H_12_O_3_	228.25	226.9	142.9	> LOQ	New in genus
Naringenin	11.86	C_15_H_12_O_5_	272.25	270.9	151.0	> LOQ	New in genus
Eriodictyol	11.38	C_15_H_12_O_6_	288.25	286.9	151.0	> LOQ	New in genus

Abbreviations: EM, experimental mass (*m*/*z*); LOQ, limit of quantification; MF, molecular formula; MS/MS, MS/MS fragments (*m*/*z*); Rt, retention time (min); TM, theoretical mass (Da).

Gallic acid is one of the most common phenolic acids, was previously documented in hydroalcoholic CHE of *M. bella* [19]. Similarly, Paganelli et al. [18] identified it in the hydroalcoholic CHE of *Myrcia splendens* leaves. Several pharmacological activities were described for this compound, as anti‐inflammatory, anti‐ulcerogenic, anticancer, antifungal, antibacterial, and antioxidant properties. In addition, it is utilized in the industry for skin applications, serving as a chelating agent [20], some of them observed for the samples tested in this research. Chlorogenic acid is an abundant phenolic compound, detected in this genus for the first time. Its pharmacological activity is closely associated with its antioxidant action, which is highly significant in the formulation of cosmetics, anti‐inflammatories, and anti‐genotoxic agents [21], and also demonstrated important anti‐acne and antiaging activity [22]. Chlorogenic acid can enhance the biosynthesis and secretion of collagen in human dermal fibroblasts (HDFs), regulating collagen and HDFs apoptosis, thereby protecting the skin against UVA radiation and preventing photoaging [23]. Another compound identified in *M. neoobscura* is *p*‐coumaric acid (4‐hydroxycinnamic acid). Due to its chemical structure being very similar to l‐tyrosine, involved in cellular melanogenesis, it can be a potent and selective inhibitor of tyrosinase, a crucial natural skin‐lightening agent to be studied. This compound facilitates cellular regeneration of wounds through its antioxidant activity [24]. *p*‐Coumaric acid was reported for the first time in the *Myrcia* genus in the crude dichloromethane extract from the leaves of the *M. splendens* [18].

Another identified phenolic compounds, such as catechin a flavanol, isoorientin (a flavone), taxifolin (flavanonol), resveratrol (a stilbene), eriodictyol and naringenin (flavanones) were reported for the first time in the *Myrcia* genus. Although these compounds could not be quantified due to their concentration being below the limit of quantification, their combined presence in the CHE can hold significant pharmacological activity as antioxidants [25].

Isoorientin may protect HDFs against the effects caused by UVB radiation, indicating a potential photoprotective action and the elimination of ROS, acting as an antioxidant [26]. Similarly, taxifolin has demonstrated photoprotective action against UVA and antioxidant properties due to its structure containing two aromatic rings, allowing it to absorb UVA (long‐wave, 320–400 nm) and UVB (medium‐wave, 280–320 nm) rays within the range of 200–400 nm [7, 27], showing photoprotective action against UVA radiation, which is more harmful to the skin as it penetrates the dermal layer [27]. Taxifolin also demonstrates anti‐inflammatory, antibacterial, angiogenesis modulator, and has potential wound‐healing properties [28].

Biological activities of resveratrol include antioxidant, anti‐inflammatory, photoprotective, anti‐scarring preventing hypertrophic scars or tissue fibrosis caused by the deposition of extracellular matrix (ECM) components and wound healing, making it an important antiaging component [29]. Although this compound is already used in cosmetic formulations [30], not all mechanisms of its contributions to the skin have been elucidated [29]. Another identified compound in this species is eriodictyol, a flavanone that has demonstrated anti‐inflammatory, antioxidant, and anti‐photoaging properties [27]. Naringenin is a flavanone that has demonstrated several activities, including antioxidant, anti‐inflammatory [31], and chronic wound‐healing agent [32].

### 2.2 In Vitro Antioxidant and Antiglycant Activities of CHE and Fractions of *M. neoobscura* Leaves

In these assays, CHE and the fractions IF, EAF, and AF were tested. Due to the lowest concentrations of total phenolic, compounds, as well as flavonoid and flavanols, HF and DF were not utilized for the subsequent biological activity assays. As observed in Table 3, in the 2,2‐diphenyl‐1‐picryl‐hydrazyl (DPPH) assay, the best results are noted for the CHE and the AF. Nevertheless, all samples exhibited excellent activity with low IC_50_ values, indicating high antioxidant activity nearing the activity of the positive control gallic acid. In the evaluation of ion Fe^+2^ chelating activity, it was observed that the AF, IF, and CHE fractions exhibited better results. Regarding the assessment of cupric ion reducing antioxidant capacity (CUPRAC), all fractions demonstrated reduction. Notably, the EAF and AF fractions displayed the highest activity. In the assessment of nitric oxide (NO) free radical scavenging capacity, all samples also demonstrated excellent results, with the better results observed for AF, that showed superior activity when compared to gallic acid. These results may be associated with the presence of the highest amount of flavanols, compared to the CHE and other fractions (Table 1). In the superoxide anion radical (^•^O^2−^) scavenging assay, IF exhibited result very close to gallic acid, used as the positive control. An interesting observation was that despite the EAF fraction did not yielding the best results in the previous tests, in the determination of ferric ion reducing potential (RP), this sample demonstrated the highest activity.

**TABLE 3:** Antioxidant and antiglycant activity of CHE and fractions of *Myrcia neoobscura* leaves.

Sample	DPPH, IC_50_ (µg mL^−1^)^a^	Chelanting Fe^2+^ (%)	CUPRAC^b^(mgGA g^−1^)	No scavenging (%)	O^−2●^ scavenging (%)	RP^c^ (mgAAc g^−1^)	Antiglycant (%)
CHE	13.61 ± 0.91a	60.12 ± 1.62a	53.99 ± 0.35a	49.08 ± 2.42a	56.65 ± 5.51a	290.9 ± 34.52a	29.72 ± 3.19a
IF	16.68 ± 3.90a,b	60.12 ± 1.73a	53.16 ± 2.69a	49.76 ± 4.19a	69.27 ± 2.42b,d	278.0 ± 7.27a	42.73 ± 1.21b
EAF	21.52 ± 2.12b	32.92 ± 2.83b	91.72 ± 0.83b	41.41 ± 1.74b	32.57 ± 2.29c	919.1 ± 155.6b	53.94 ± 1.90c
AF	9.50 ± 0.47a	61.04 ± 0.43a	74.84 ± 2.33c	57.15 ± 1.33c	61.62 ± 2.16a,b	415.8 ± 27.99a	45.02 ± 1.26b
C+^d^	1.52 ± 0.08c	ND	ND	40.31 ± 2.21b	73.39 ± 3.17d	ND	56.43 ± 1.47c

*Note*: All tests were performed in triplicate with test sample concentration of 1000 µg mL^−1^. The results are expressed as media ± SD of three independent experiment. Statistical analysis was performed using one‐way ANOVA followed by Tukey's test, results with equal letters did not differ statistically (*p* ≤ 0.05).

Abbreviation: ND, non‐detected.

^a^ IC_50_ = sample concentration necessary to inhibit 50% of DPPH radical (µg mL^−1^).

^b^ Cu^2+^ = chelating effect, expressed in mg of gallic acid per dry extract (mgGA g^−1^).

^c^ RP = reducing potential of ferric ion, expressed in mg of ascorbic acid per dry extract (mgAAc g^−1^).

^d^ C+ = positive control: gallic acid (1000 µg mL^−1^), for DPPH, O^•−^ and NO scavenging assay; EDTA 100 µg mL^−1^) for chelating Fe^2+^ assay; quercetin (100 µg mL^−1^) for antiglycant assay.

A study with *Myrcia* spp. [33] indicates that the species with a higher content of phenolic compounds, such as phenolic acids tend to exhibit greater antioxidant activity. Studies [15, 34] also report that antioxidant activity is associated with the composition of phenolic compounds, including phenolic acids and flavonoids. In the present study, a similar pattern was observed; the samples with a higher quantity of phenols and flavanols demonstrated greater antioxidant activity (as seen in AF and CHE).

The importance of searching for natural antioxidants, such as phenolics, has been recognized due to their potential action in eliminating and suppressing the formation of ROS or both, opposing their actions, what is associated with oxidative cellular damage, the imbalance between the generation of free radicals and primary cause of cardiovascular diseases, cancer, and aging. These natural antioxidants exert their actions through different properties such as free radical scavenging, hydrogen donors, reducing agents, singlet oxygen quenchers, and metal chelators [15].

The study by Lima Neto et al. [35] reinforces the presence of flavonoids and phenolic acids with antioxidant activity, playing a role in scavenging ROS, reducing, and chelating ferric ions that catalyze lipid peroxidation, impacting skin aging. In the test of reducing capacity for Fe^3+^ and Cu^2+^, the result of *M. neoobscura* leaves may be related to the ability of these phenolic compounds to chelate these transition metals. This test is important as it demonstrates that the extract or fraction has the capability to interrupt the reaction of free radicals in lipid oxidation, altering the redox potential of the environment, and repairing molecules damaged by free radicals [36]. Free iron and copper are generally bound to free radicals through the Fenton and Haber–Weiss reactions. Flavan‐3‐ols (catechin) bind to these divalent transition metals, effectively reducing their concentration and thus attenuating the extent of oxidative activity [22]. This chelating capacity for copper also influences tyrosinase activity since the active site of tyrosinase contains copper [37]. The antioxidant activity of the plant may be interconnected with the tyrosinase inhibition test.

Regarding the antiglycant activity, these are the first results for *M. neoobscura* species. As shown in Table 3, the antiglycation activity demonstrated excellent results in the EAF fraction, compared to the positive control quercetin. The advanced glycation end‐products (AGEs) are heterogeneous products resulting from the reaction of reducing sugars and reactive aldehydes with proteins, lipids, and nucleic acids. This nonenzymatic reaction occurs slowly in tissues, involving glycation and oxidation processes. Long‐lived proteins, such as elastin and dermal collagen are susceptible to these modifications. Another factor is that AGEs tend to accumulate primarily in the dermis of aged skin rather than in the epidermis due to its rapid and constant renewal. This process can be accelerated in conditions of hyperglycemia and the presence of oxidative stress. AGEs also play a role in photoaging, causing the accumulation of abnormal elastic fibers, apoptosis, and senescence of dermal fibroblasts. They also stimulate inflammation by binding to the receptor for AGEs (RAGE). Exposure to UV radiation can increase the production and secretion of these molecules from keratinocytes, reducing the activity of antioxidant enzymes and degrading AGEs, which can directly promote melanin synthesis, contributing to the hyperpigmentation associated with photoaging [38].

### 2.3 Enzymatic Inhibitory Activity and Photoprotective Potential and of CHE and Fractions

The samples exhibited significant collagenase inhibitory activity, with IC_50_ values closely comparable to that of the positive control, gallic acid (IC_50_ = 108.88 ± 12.71 µg mL^−1^). AF showed IC_50_ values (89.75 ± 6.68 µg mL^−1^) approximately 20% higher than gallic acid, and the differences were still statistically significant, underscoring their strong inhibitory potential in this assay. Similar to collagenase‐inhibiting activity, the samples were notable elastase inhibitors. The enzyme was particularly affected by CHE (IC_50_ = 317.97 ± 2.57 µg mL^−1^) and AF (IC_50_ = 351.19 ± 4.37 µg mL^−1^), whose activity did not differ from the activity of the employed standard inhibitor, ursolic acid (IC_50_ = 332.67 ± 38.16 µg mL^−1^).

As the skin ages, levels of elastin and collagen progressively decline, leading to visible signs of aging such as wrinkles and sagging [39]. Elastin is a key ECM protein that provides tissues with the ability to undergo elastic recoil, a property essential for the structural integrity of the skin, as well as organs such as arteries, lungs, and ligaments. In the skin, elastin contributes to maintaining elasticity and resilience; however, it is susceptible to degradation by elastase, a serine protease that acts within the ECM. Several studies have shown that human neutrophil elastases are also involved in the degradation of ECM connective tissue through the activation of inactive MMP‐1 and MMP‐2 precursors (matrix metalloproteinases [MMPs]), induced by UV radiation and ROS imbalance [40]. Collagen is the primary structural protein of the ECM and plays a crucial role in maintaining the skin's strength, firmness, and flexibility. The enzyme collagenase is responsible for ECM remodeling by cleaving specific peptide bonds within the collagen triple helix, thereby contributing to collagen degradation and tissue turnover [39].

All samples exhibited inhibitory activity against tyrosinase; however, the inhibition levels were lower than those observed for the reference compound, kojic acid (IC_50_ = 5.03 ± 0.03 µg mL^−1^). Among the tested samples, IF (IC_50_ = 98.11 ± 1.16 µg mL^−1^) demonstrated the highest tyrosinase‐inhibitory activity, followed by AF (IC_50_ = 120.65 ± 8.28 µg mL^−1^). Tyrosinase is a multifunctional enzyme that plays a pivotal role in melanin formation. Its distribution spans melanosomes in fungi, mammals, bacteria, and plants. In mammals, it contributes to melanin synthesis through the melanogenesis pathway, wherein the oxidation of tyrosine to dopaquinone marks the initial step. Unregulated tyrosinase activity can lead to skin hyperpigmentation. To counteract this, synthetic or natural tyrosinase inhibitors, for example, plant secondary metabolites, particularly phenolic compounds such as flavonoids, constitute the most extensive group of inhibitors [5].

Most of the extracts exhibited some lipoxygenase (LOX) inhibitory activity, with the exception of IF, which showed no inhibition and was therefore excluded from the corresponding figure. Among the active samples, CHE (IC_50_ = 74.15 ± 0.51 µg mL^−1^) demonstrated the strongest LOX inhibition; however, its activity remained lower than that of the standard inhibitor, nordihydroguaiaretic acid (NDGA) (IC_50_ = 5.44 ± 0.02 µg mL^−1^).

Numerous studies have demonstrated that phenolic compounds possess inhibitory effects on key enzymes associated with skin aging, including collagenase, elastase, and tyrosinase. These enzymes play pivotal roles in the degradation of the ECM and melanin synthesis, processes that contribute to skin aging and hyperpigmentation. The phytochemical profiling of *M. neoobscura* via HPLC–ESI–MS/MS revealed the presence of significant phenolic constituents, notably flavonoids, catechins, phenolic acids, and stilbenes. Such compounds are recognized for their bioactive properties, including antioxidant activity and enzyme inhibition, which are beneficial in cosmetic and dermatological applications.

**FIGURE 2:**
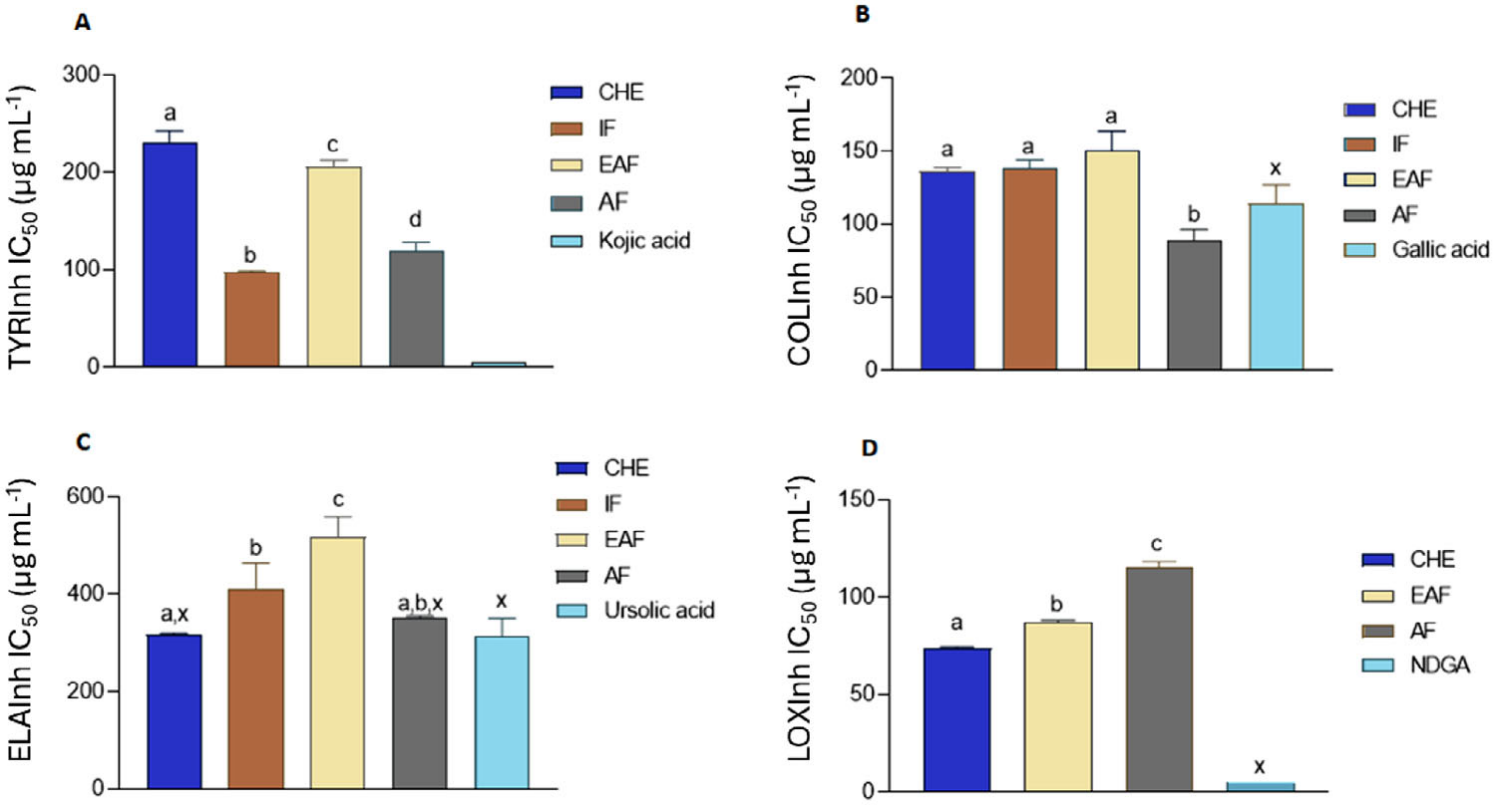
Enzymatic inhibitory activity of CHE and fractions of *Myrcia neoobscura*. (A) Tyrosinase; (B) collagenase; (C) elastase; (D) lipoxygenase. IC_50_ = sample concentration necessary to inhibit 50% of tyrosinase activity in µg mL^−1^. Positive controls: kojic acid (1000 µg mL^−1^, in methanol), for tyrosinase; gallic acid, for collagenase; ursolic acid, for elastase and nordihydroguaiaretic acid (NDGA) on lipoxygenase. Results with equal letters did not differ statistically (*p* ≤ 0.05). *x* = difference with the positive control (one‐way ANOVA followed by Dunnett's posttest, *p* < 0.05). Samples not connected with the same letter are statistically different.

Polyphenols, such as catechin and epigallocatechin gallate, due to the presence of catechol moiety [41], may inhibit the activity of proteolytic enzymes in vitro by acting as complexing or precipitating agents and have been shown to exhibit moderate inhibitory effects against collagenase and elastase activity, presumably through non‐covalent binding [42]. Protocatechuic acid, a phenolic acid found in the *M. neoobscura* extract, demonstrated inhibitory effects on tyrosinase, collagenase, elastase, and hyaluronidase enzymes, in addition to increasing Type I collagen levels and inhibiting MMP‐1 [43]. In addition, the free carboxyl moiety in gallic acid could bind to the zinc site in the catalytic domain of collagenase as possible inhibition mechanism (Figure 2).

Furthermore, due to the presence of the hydroxyl group in their structure, polyphenols are considered the largest group of tyrosinase inhibitors. Flavonoids play a crucial role in reducing skin hyperpigmentation as they exhibit anti‐tyrosinase activity through some pathways, such as competitive inhibition (related to the structural similarity of the amino acid tyrosine) and noncompetitive inhibition. In addition, they negatively regulate MITF expression and suppress the cAMP‐binding protein element pathway (cAMP‐CREB) [11].

Among the phenolic acids [44], a study demonstrating that *p*‐coumaric acid is a mixed‐type or competitive inhibitor of tyrosinase, depending on the substrate used: l‐tyrosine or l‐DOPA. Due to the structural similarity to the substrate l‐tyrosine, this compound can bind and block the enzyme active site, preventing the access of the substrate, which can explain, in part, the observed anti‐tyrosinase activity. Another important point to highlight is that both the CHE and the fractions showed excellent antioxidant activity. The chelating capacity of Cu^2+^ (CUPRAC) may be related to tyrosinase activity, due to the presence of Cu^2+^ in the tyrosinase active site [37].

Regarding the sun protection factor (SPF) values of CHE and fractions of *M. neoobscura*, best SPF values were found in CHE, EAF, and IF at a concentration of 250 µg mL^−1^ with SPF of 14.16 ± 0.83, 13.91 ± 0.93, and 12.82 ± 0.96, respectively. The photoprotective efficacy of sunscreens is assessed based on SPF, being the standard measure in the sunscreen industry. The SPF values indicate the protective capacity primarily against UVB light, but this alone is insufficient to assess the total amount of UV radiation penetrating the skin. It directly quantifies protection against sunburn, known as UV‐induced skin erythema, primarily caused by UVB exposure [13]. The phenolic compounds identified by HPLC–ESI–MS/MS (Table 2), including gallic acid, chlorogenic acid, *p*‐coumaric acid, such as protocatechuic acid, isoorientin, taxifolin, resveratrol, naringenin, and eriodictyol exhibit significant photoprotective activity [23, 26, 27, 29, 45, 46, 47, 48, 49]. These compounds demonstrate the ability to absorb both UVA and UVB radiation. UVB radiation is considered the inducer of tissue damage caused by irradiation, leading to cellular death, particularly in HDFs, resulting in collagen accumulation, fibrosis generation, and intradermal skin remodeling, ultimately causing the development of thick wrinkles. The generation of ROS leads to the degradation of ECM components through increased production of MMPs such as MMP‐1 and MMP‐3, contributing to skin damage and photoaging. Isoorientin is a flavonoid, characterized by the presence of more than one benzene ring in their chemical structure, which imparts the ability to absorb UV radiation [26]. Protocatechuic acid also provides protection against UVB by absorbing and restoring redox balance disrupted by ROS [48]. Chlorogenic acid and taxifolin are compounds capable of absorbing UVA radiation. UVA photons can penetrate the skin deeply, causing damage to both the epidermis and dermis through the generation of ROS. Taxifolin inhibits ROS, glutathione depletion, formation of single‐strand breaks, and activation of caspase‐3 in human dermal HDFs. Chlorogenic acid increased collagen metabolism of Col1 type, regulated MMP levels such as MMP‐1 and MMP‐3, reduced ROS formation and DNA damage, increased cell repair, and decreased HDFs apoptosis [23].

### 2.4 Evaluation of In Vitro Wound‐Healing Activity of CHE Using Scratch Assay and Cell Viability on L929 Fibroblast

**FIGURE 3:**
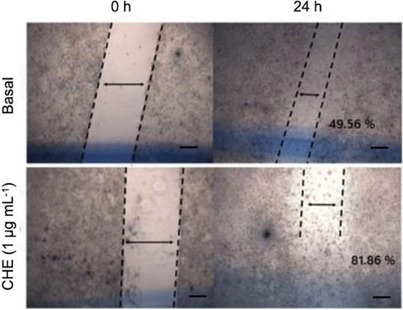
Effect of CHE from *Myrcia neoobscura* leaves on fibroblast (L929) migration. Image representation of fibroblast migration (L929) in vitro scratch. The results were expressed as mean. Scale bar = 200 µm.

Figure 3 illustrates that, within 24 h, fibroblasts treated with CHE exhibited migration into the skin tear, yielding an excellent outcome of 81.86% compared to the baseline without any treatment (49.56%). This result holds significant importance, particularly considering the polyphenolic nature of the *Myrcia* genus, and is the first investigation into the healing activity of *M. neoobscura*. Literature suggests [50] that flavonoids can potentially impact the wound‐healing process, influencing collagen degradation and MMP‐2 activity after 24 h. The notable activity observed in *M. neoobscura* CHE may be attributed to the substantial presence of flavonoids.

The epidermis, the outermost skin layer, primarily composed of keratinocytes, forms the initial barrier against external factors. Anchored by hemidesmosomes, collagen XVII, laminin 332, and integrin α6β4 to the basement membrane (BM), it regulates molecular exchange between the epidermis and dermis, aiding keratinocyte and fibroblast adhesion, migration, and proliferation, crucial for wound re‐epithelialization [51, 52]. The dermis, beneath the epidermis, comprises connective tissue with fibroblasts as the primary cell type, secreting collagen, elastin, and other ECM components, providing skin elasticity and resistance [53]. Aging leads to decreased fibroblast activity, reducing collagen, elastin, and hyaluronic acid synthesis, exacerbated by increased enzyme activity like collagenases, contributing to skin aging. Furthermore, photodamage intensifies collagen loss [54, 55].

Natural bioactive compounds with healing properties have garnered attention for their potential to enhance the innate immune response, fostering cellular protection and differentiation of keratinocytes and dermal fibroblasts. In addition, they may play a pivotal role in collagen synthesis, thereby influencing the wound‐healing process positively. Plants rich in polyphenols, known for their anti‐inflammatory, antioxidant, and antimicrobial properties, are particularly promising in promoting epithelialization and facilitating wound healing [56]. Among the phenolic compounds observed in the CHE by HPLC–ESI–MS/MS, resveratrol was identified, which has been reported as a wound‐healing agent. Resveratrol protects fibroblasts by acting as an antioxidant, reducing ROS formation, stabilizing cell proliferation, improving cell migration quality, and preserving structural integrity. In addition to demonstrating wound‐healing properties, resveratrol also helps prevent excessive scarring, serving as an antioxidant agent [29]. Furthermore, Trinh et al. [57] report that gallic acid can stimulate keratinocyte migration during wound‐healing and activate healing factors such as c‐jun N‐terminal kinases, focal adhesion kinases, and extracellular signal‐regulated kinases. Naringenin, another flavonoid characterized in this study, is known for reducing ROS production and inflammation, and catechin, which also reduces ROS production and modulates macrophage polarity to aid in wound healing [32]. Due to these compounds being present in the CHE and showing excellent antioxidant action, the potential wound‐healing effect on fibroblasts of polyphenols can once again be related.

### 2.5 Evaluation of Toxicity of CHE and Fractions From *M. neoobscura*

**FIGURE 4:**
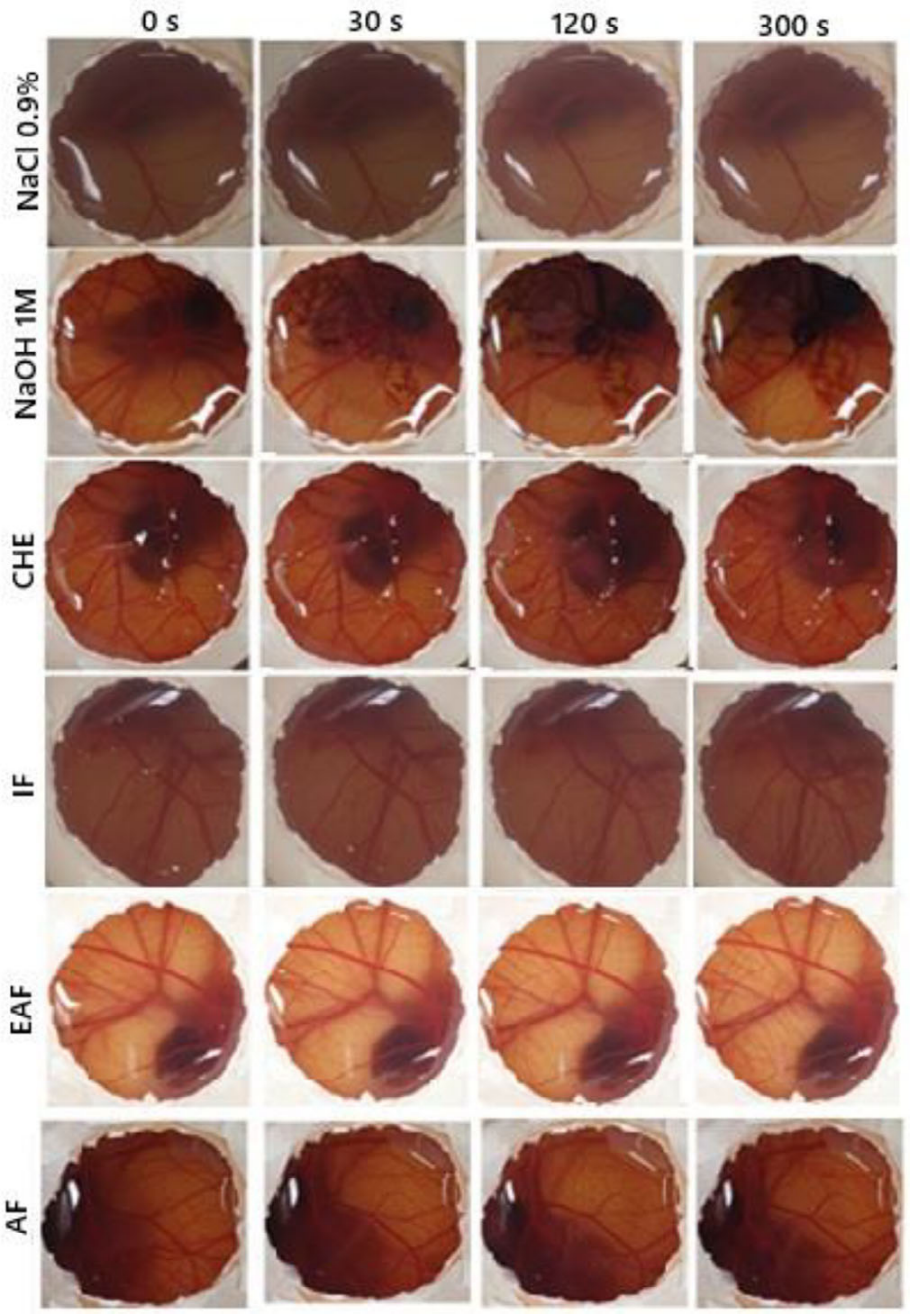
Irritation of *Myrcia neoobscura* CHE and fractions by HET‐CAM. In the HET‐CAM assay the CAM was exposed to *M. neoobscura* (300 µg mL^−1^) extract and fractions for 5 min for hemorrhage, vessel lysis or coagulation observation. AF, aqueous fraction; CHE, crude hydroalcoholic extract; EAF, ethyl acetate fraction; IF, insoluble fraction; NaOH (1 M)*: positive control; NaCl 0.9%: negative control.

The CHE from *M. neoobscura* leaves were tested for toxicity by the MTT assay in fibroblasts (L929). No cytotoxic effects were detected when L929 cells were cultivated with different concentrations of 1 at 100 µg mL^−1^, making it a safe alternative for use for cosmetic purposes. By the HET‐CAM method (Figure 4), irritation of CHE and fractions was analyzed at 30, 120, and 300 s, such as vascular lysis, hemorrhage, and coagulation (Table 4).

**TABLE 4:** Toxicity assessment of CHE and fractions of *Myrcia neoobscura* using the HET‐CAM method.

Sample	In vitro score sum	Result
CHE	1.66	Light irritating
IF	1	Light irritating
EAF	2	Light irritating
AF	0	Non irritating

**FIGURE 5:**
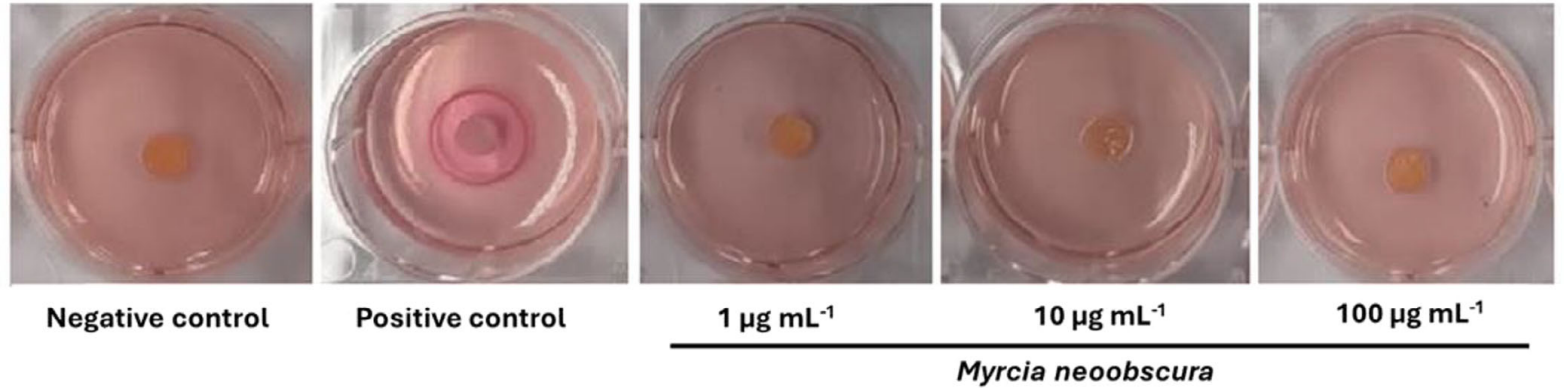
Irritation of *Myrcia neoobscura* CHE and fractions by agarose overlay assay. Ilustrative figure representing agarose overlay assay in murine fibroblast cell line L929. Negative control: PBS; positive control: SDS; CHE of *M. neoobscura* was tested at 1, 10, and 100 µg mL^−1^. The plates were incubated 24 h at 37°C with 5% CO_2_. The degree of irritation was evaluated by lysis zone (lack of incorporation of the vital dye).

CHE, IF, and EAF showed slight irritation and AF showed no irritation. In the agarose‐overlay assay using murine fibroblast cell line L929, CHE at concentrations of 1, 10, and 100 µg/mL (Figure 5) did not induce cell lysis, even at the highest concentration tested. This outcome was comparable to the negative control PBS. In contrast, the positive control (sodium dodecyl sulfate [SDS]) produced a clear lysis halo surrounding the application disc, lacking vital dye uptake (neutral red). These results are consistent with those obtained from the MTT and HET‐CAM assays, reinforcing the safety profile of CHE for topical applications. Moreover, in vitro scratch assay data support its potential wound‐healing capacity, suggesting promising phytocosmetic applicability in skin care formulations.

## 3 Conclusions

The phytochemical characterization of *M. neoobscura* leaves showed that the hydroalcoholic extract (CHE) and its fractions contain significant amounts of polyphenolic compounds, which contribute to antioxidant, anti‐glycation, anti‐collagenase, anti‐elastase, anti‐LOX, anti‐tyrosinase, photoprotective activities, and wound‐healing properties. Phytochemical analysis of CHE by HPLC–ESI–MS/MS revealed the presence of phenolic compounds, such as phenolic acids and cinnamic acids, in addition to flavonoids, which may be related to the described activities. Due to their antioxidant capacity, the samples inhibit the formation of AGEs, which may prevent the degradation of collagen and elastin and inhibit degradation enzymes. In addition, they provide photoprotection and may reduce the free radicals induced by sun exposure. The wound‐healing assay with murine fibroblasts (L929), using the in vitro scratch method, showed an effectiveness of 81.86% for CHE. Regarding toxicity, samples did not show cytotoxicity in the MTT, or the agarose overlay assays, with slight irritation observed in the HET‐CAM test. This study contributes to the understanding of the composition of this novel species, demonstrating its significant biological activity. The results from the CHE and its fractions suggest their potential as phytocosmetic agents.

## 4 Experimental Section

### 4.1 Collection and Identification of Plant Material

The leaves were collected in May 2022, in Blumenau, SC, in São Francisco Park (26°55′22″ S; 49°4′39″ W). The identification of plant material was carried out by botanist Prof. Dr. André Luis de Gasper, FURB). A sample was deposited in the Dr. Roberto Miguel Klein Herbarium under number 72435. The project is registered in SISGEN under number A7519AA. After collection, leaves were dried and grounded in a knife mill, yielding 840.3 g. The plant material was macerated in ethanol 70% within 7 days (twice), filtered and concentrated in a rotary evaporator under reduced pressure at a temperature below to 60°C until obtaining the CHE (106.4 g). An aliquot of 55 g CHE was resuspended in ethanol 20%, storage under low temperature (2°C–8°C) for 12 h and filtered. The resulting precipitate was separated from the sample to afford the IF (8.5 g). The supernatant was partitioned in solvents of increasing polarity hexane > dichloromethane > ethyl acetate), yielding hexane (HF) (0.3 g), dichloromethane (DF) (0.2 g), EAF (1.2 g), and finally AF (44.8 g).

### 4.2 Quantitative Assessment of Secondary Metabolites in the Extract and Fractions

#### 4.2.1 Quantification of Total Phenolic Contents

The quantification of TPCs was carried out using the Folin–Ciocalteu reagent, as proposed by Anagnostopoulou et al. [58], with slight modifications. The concentration of TPC was estimated by interpolation using the calibration curve performed with standard solutions of gallic acid (*y* = 0.007*x* + 0.0445; *r* ^2^ = 0.9989). The content of TPCs was expressed in mg of gallic acid per g of extract and fractions (mgAG g^−1^ of the extract and fractions). Tests were performed in triplicate.

#### 4.2.2 Quantification of Total Flavonoid Contents

For the quantification of flavonoids, the methodology proposed by Woisky and Salatino [59] was employed. To calculate the flavonoid content, a calibration curve was constructed using quercetin (*y* = 0.0067*x* + 0.0618; *r* ^2^ = 0.9975). The content of flavonoids was expressed as mg of quercetin per g of extract (mgQUE g^−1^ of the extract). Tests were performed in triplicate.

#### 4.2.3 Quantification of Total Flavanol Content

The content of total flavanol compounds was determined by the colorimetric method of vanillin with catechin as a standard, as proposed by Jayaprakasha et al. [60]. The concentration of total flavanol was estimated by a calibration curve constructed with standard catechin solutions (*y* = 0.0005*x* + 0.0089; *R* ^2^ = 0.9983). To eliminate the interference of the extract, a blank solution was prepared for each sample using a 4% HCl solution in MeOH instead of the vanillin reagent. Total flavanol content was expressed as mg of catechin equivalent per g of extract (mgCAT g^−1^).

#### 4.2.4 Analysis of Phenolic Compound by HPLC–ESI–MS/MS

The HPLC–ESI–MS/MS analysis was performed using the sample pretreatment, chromatographic, and mass spectrometer parameters previously described by Borges et al. [61]. The eluent was formed by mixing Solvents A (MeOH/H_2_O in ratio of 95%) and B (H_2_O/formic acid 0.1%) as follows: first stage—10% Solvent A and 90% B (isocratic mode) for 5 min; second stage—linear gradient of Solvents A and B (from 10% to 90% of A) for 2 min; third stage—90% Solvent A and 10% B (isocratic mode) for 3 min; fourth stage—linear gradient of Solvents A and B (from 90% to 10% of A) for 7 min, with a flow rate of 250 µL min^−1^ of mobile phase. In all analyses, the injected volume was 5 µL. Samples were prepared by dissolving 50 mg of dried material in a 5 mL solution of hydrochloric acid at pH 2. These solutions were extracted three times with 2 mL of ethyl ether and the ethereal extract resulting of three extractions of each sample were combined, dried and stored in a sealed container at −20°C. Before analysis, the dried material was dissolved in 1 mL of MeOH and centrifuged at 14 000 rpm for 5 min, being dissolved in 10 parts of ultrapure water.

For the identification and quantification, 39 standard phenolic compounds were analyzed under the same conditions described above, being them: abscisic acid, 4‐aminobenzoic acid, 4‐hydroxymethylbenzoic acid, apigenin, caffeic acid, catechin, chlorogenic acid, chrysin, cinnamic acid, coniferaldehyde, eriodictyol, ferulic acid, fustin, gallic acid, hispidulin, isoorientin, isoquercetin, mandelic acid, methoxyphenylacetic acid, naringerin, *p*‐anisic acid, *p*‐coumaric acid, pinocembrin, protocatechuic acid, resveratrol, rosmarinic acid, rutin, salicylic acid, scopoletin, sinapaldehyde, sinapic acid, syringaldehyde, syringic acid, taxifolin, umbelliferone, 4‐methylumbelliferone, vanillic acid, vanillin, and vitexin dissolved in methanol (0.02–6 mg L^−1^) [61], according to the analytical parameters of the chromatographic method shown in Table 5.

**TABLE 5:** Performance parameters of the chromatographic method for the determination of phenolic compounds of *Myrcia neoobscura* leaves.

Phenolic compound	LOD (mg L^−1^)	LOQ (mg L^−1^)	Linear regression equation	*R* ^2^
Gallic acid	0.01	0.02	*y* = 293770*x* − 7204	*R* ^2^ = 0.999
Protocatechuic acid	0.01	0.03	*y* = 47122*x* − 204	*R* ^2^ = 0.998
Catechin	0.03	0.08	*y* = 173788x+9289	*R* ^2^ = 0.998
Chlorogenic acid	0.02	0.08	*y* = 200305*x* + 21347	*R* ^2^ = 0.997
Isoorientin	0.01	0.04	*y* = 54348*x* + 850	*R* ^2^ = 0.997
*p*‐Coumaric acid	0.14	0.43	*y* = 6406x ‐2181	*R* ^2^ = 0.978
Taxifolin	0.05	0.16	*y* = 630796*x* − 62958	*R* ^2^ = 0.995
Resveratrol	0.01	0.04	*y* = 355620*x* − 4184	*R* ^2^ = 0.998
Eriodictyol	0.06	0.2	*y* = 4251*x* − 223	*R* ^2^ = 0.998
Naringenin	0.01	0.02	*y* = 455652*x* + 9980	*R* ^2^ = 0.999

*Note*: LOD and LOQ were obtained from the signal–noise ratio according to Ribani et al. [62].

Abbreviations: LOD, limit of detection; LOQ, limit of quantification; *R* ^2^, coefficient of determination.

### 4.3 Evaluation of In Vitro Antioxidant Activity

#### 4.3.1 Determination of DPPH Free Radical Scavenging Activity

The antioxidant activity of the free radical DPPH was determined according to Cavin et al. [63], with samples diluted in methanol (7.81–1000 µg mL^−1^). BHT was used as a standard in the same concentrations. The potential DPPH radical scavenging activity (%) was calculated using the following formula (*A*_s_/*A*_c_) × 100, where *A*_s_ is the absorbance in the presence of the sample and *A*_c_ is the control absorbance. The test was carried out in triplicate, and the results were expressed as IC_50_ (concentration necessary to inhibit 50% of the DPPH radical, µg mL^−1^) in the form of mean ± standard deviation (SD).

#### 4.3.2 Determination of the RP of Ferric Ion

Analysis of RP of ferric ion was based on the method proposed by Waterman and Mole [64]. In the test tube, 50 µL of samples (1000 µg mL^−1^ diluted in MeOH), 4.25 mL of distilled water, and 0.5 mL of FeCl_3_ 0.1 M were added. After 3 min, 0.5 mL of potassium ferricyanide 0.008 M was added and left at room temperature for 15 min. The absorbances of the samples were determined at 720 nm. The blank solution was prepared using methanol instead of the sample according to the procedure above. The experiment was carried out in triplicate. The RP of ferric ion was estimated by interpolation on a calibration curve constructed with solutions standard ascorbic acid (*y* = 0.002*x* + 0.0191; *r* ^2^ = 0.9996). The RP of the samples was expressed in mg of ascorbic acid per gram of extract (mgAAc g^−1^).

#### 4.3.3 Nitrogen Free Radical (NO) Scavenging Capacity

The nitrogen free radical scavenging activity of the extracts was determined using the NO radical scavenger assay, as described by Moe et al. [65]. The reaction mixture, containing 100 µL of test samples at different concentrations (31.25–1000 µg mL^−1^ in methanol), 200 µL of PBS solution (0.1 M, pH 7.4), and 700 µL of sodium nitroprusside 10 mM (prepared in 0.1 M phosphate buffer solution, pH 7.4), was incubated at 25°C for 90 min to allow the formation of nitrite ions. After incubation, 500 µL of 0.33% sulfanilic acid reagent (prepared in 20% glacial acetic acid) was added, and the mixture was left at room temperature for 5 min to complete the diazotization. Afterward, 500 µL of 0.1% *N*‐(1‐naphthyl)ethylenediamine dihydrochloride (prepared in 20% glacial acetic acid) was added. After 20 min, the absorbance of the pink chromophore was measured in a spectrophotometer at 540 nm. A negative control was prepared using methanol in place of the sample. A blank solution using methanol in place of nitroprusside sodium was prepared. Gallic acid was used as a standard. The rate of inhibition of the formation of NO radicals was calculated using the formula inhibition NO (%) = Absorbance_negative control_ − Absorbance_sample_/Absorbance_negative control_ × 100. Tests were performed in triplicate.

#### 4.3.4 Chelating Potential on Ferrous Ion

The chelating capacity of ferrous ions was assessed using the method described by Kilic et al. [66]. A reaction mixture was prepared, comprising 0.4 mL of samples at varying concentrations (31.25–1000 µg mL^−1^) diluted in methanol, 3 mL of distilled water, 0.1 mL of 0.6 mM FeCl_2_ solution, and 0.1 mL of 5 mM ferrozine solution. The mixture underwent a 10‐min incubation at room temperature, and the absorbance of the solutions was measured at 562 nm. Methanol served as a substitute for the sample in the negative control. A blank solution was prepared using a sample solution and methanol instead of the reagents. The ferrous ion chelating potential was calculated according to the formula: potential Fe^2+^ (%) = [1 − *A*_1_ − *A*_b_) − *A*_0_)] × 100, where *A*_0_ is the absorbance of the negative control, *A*_1_ and *A*_b_ are the absorbances of the sample and blank solution, respectively. Tests were performed in triplicate.

#### 4.3.5 In Vitro Superoxide Radical (•O^2−^) Scavenging Assay

The assay had been determined using the nitro blue tetrazolium (NBT)/riboflavin reaction [65]. The reaction mixture, containing 100 µL of the test samples (1 mg mL^−1^ in methanol), 1.6 mL of PBS (pH 7.4), 0.15 mL of EDTA (4.5 mmol L^−1^), 0.10 mL of NBT (1 mg mL^−1^), and 0.05 mL of riboflavin (0.2 mg mL^−1^), was incubated for 5 min under fluorescent light. The absorbance of the solution was measured at 560 nm against the corresponding negative control (substituting samples with methanol). Gallic acid was used as a standard. The inhibition rate was calculated using the formula: inhibition (%) = [Absorbance_negative control_ − Absorbance_sample_)/Absorbance_negative control_] × 100. Tests were conducted in triplicate.

#### 4.3.6 CUPRAC

The CUPRAC method was performed as described by Apak et al. [67] with light modifications. An aliquot of 0.5 mL of CuCl_2_ solution, 0.5 mL of neocuproine solution, 0.5 mL of ammonium acetate (NH_4_Ac), buffer at pH 7.0 and 0.5 mL of test solutions (0.1 mg mL^−1^ in methanol) were mixed. The tubes were incubated at room temperature and after 30 min, and the absorbance at 450 nm was recorded against a negative control (test performed without the presence of any antioxidant). The cupric ion reducing capability was estimated by interpolation of a gallic acid calibration curve (6.25–100 µg mL^−1^, diluted in methanol, *y* = 0.0164*x* + 0.2476, *r* ^2^ = 0.9749) and it was expressed as mg of gallic acid per gram of dry extract or fraction (mgGA g^−1^ extract). The tests were performed in triplicate.

#### 4.3.7 Determination of Antiglycant Activity

The antiglycant activity was determined through the oxidative method [68]. Solutions of glyoxal (30 mM) and bovine serum albumin (BSA) (10 mg mL^−1^) were prepared in PBS (0.2 M, pH 7.4) containing 3 mM sodium azide as an antimicrobial agent. The reactions were carried out with 300 µL of the reaction mixture total (BSA) (135 µL), glyoxal (135 µL), DMSO or sample (30 µL, 1.0 mg mL^−1^ in DMSO), and incubated at 37°C for 7 days. Following incubation, each sample was analyzed in a fluorescence spectrophotometer (excitation at 330 nm and emission at 420 nm). Quercetin (100 µg mL^−1^) was used as a standard, and DMSO as a negative control. The results were expressed as a percentage of inhibition and IC_50_. The tests were conducted in triplicate.

#### 4.3.8 Tyrosinase Inhibitory Activity

Tyrosinase inhibitory activity was determined according to Jabłonowska et al. [69]. The solutions of the samples (80 µL) and freshly prepared tyrosinase (40 µL) in phosphate buffer (16 mM, pH 6.8) were combined. The mixture was left at room temperature in the dark for 10 min, where upon l‐DOPA solution 0.19 mg mL^−1^ in phosphate buffer (80 µL) was added. After an additional 10 min, the absorbance at 492 nm was measured and the tyrosinase inhibitory activity (Tylnh) was calculated as described in the Equation (1):

(1) $$\mathrm{TYRlnh}\left(\%\right)=\left(A_{0}\;-\;A_{s}\right)/A_{0}\;\times \;100$$

where *A*_0_ is the absorbance of the negative control (where buffer was used instead of the extract) and *A*_s_ is the absorbance of the respective sample. Kojic acid was used as positive control.

#### 4.3.9 Collagenase Inhibitory Activity

For collagenase inhibitory activity [70], the sample solutions (40 µL) and collagenase solution (20 µL, 1 mg mL^−1^), previously dissolved in Tris‐HCl buffer (0.1 M, pH 7.5, 5 mM CaCl_2_, and 1 µM ZnCl_2_), were combined, and the mixture kept at room temperature for 5 min. Following that, gelatin dissolved in the same buffer (40 µL, 3.44 mg mL^−1^) was added. The resulting mixture was incubated at 37°C for 40 min, whereupon the stop reagent, containing 12% (w/v) PEG 6000 and 25 mM EDTA, as well as 90 µL of the ninhydrin reagent were added. The reaction mixture was then heated for 15 min at 80°C. The ninhydrin reagent was prepared by mixing SnCl_2_ solution (80 mg of SnCl_2_·2H_2_O dissolved in 50 mL of 0.2 M, pH 5.0 citrate buffer) with an equal volume of 5% ninhydrin solution in DMSO. The collagenase inhibitory activity (COLlnh) was calculated using the equation:

$$\mathrm{COLlnh}\left(\%\right)=\left(A_{0}\;-\;A_{s}\right)/A_{0}\;\times \;100$$

where *A*_0_ is the absorbance of the negative control (water) and A_s_ is the absorbance of the respective sample. Gallic acid was used as positive control.

#### 4.3.10 Elastase Inhibitory Activity

For elastase inhibitory activity [71], the solutions of the sample (100 µL) in Tris‐HCl buffer (0.1 M, pH 8.0) were mixed with elastase solution (25 µL, 0.05 mg mL^−1^ in the same buffer) and kept for 5 min at room temperature. Thereupon a phosphate buffer saline solution of SANA (*N*‐succinyl‐(Ala)_3_‐nitroanilide) (70 µL, 0.41 mg mL^−1^) was added. The absorbance (410 nm) of the resulting mixture was measured after 40 min. Elastase inhibitory activity (ELAlnh) was calculated using the equation:

$$\mathrm{ELAlnh}\left(\%\right)=\left(A_{0}\;-\;A_{s}\right)/A_{0}\;\times \;100^{\prime }$$

where *A*_0_ is the absorbance of the negative control and *A*_s_ is the absorbance of the solution containing the respective sample.

#### 4.3.11 LOX Inhibitory Activity

LOX inhibitory activity was measured according to Chekir et al. [72]. Phosphate buffer (50 µL, 100 mM, pH 8,) was added to the mixture of LOX (25 µL, 0.0032 mg mL^−1^), and the samples (100 µL), previously dissolved in the same buffer. To the resulting mixture, 50 µL of linoleic acid solution in the same buffer was added after 5 min and the mixture was then incubated at 25°C for 15 min. The absorbance was measured at 234 nm. LOX inhibitory activity (LOXlnh) was calculated as in the equation:

$$\mathrm{LOXInh}\left(\%\right)=\left(A_{0}\;-\;A_{s}\right)/A_{0}\;\times \;100$$

where *A*_0_ is the absorbance of the negative control (reaction mixture containing buffer solution instead of the extract) and *A*_s_ is the absorbance of the respective sample.

#### 4.3.12 Determination of In Vitro Photoprotective Activity

The CHE and fractions of *M. neoobscura* were evaluated using the spectrophotometric method [73]. An absorption scan of the samples was carried out in a spectrophotometer between wavelengths of 200–800 nm to visualize the maximum absorption peaks. For the calculation of the SPF, the absorption between 290–320 nm was checked every 5 nm. SPF was calculated using the formula:


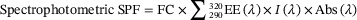

where FC is the correction factor (= 10), determined according to two SPF solar filters known, such that a cream containing 8% homosalate (4 SPF); EE(λ) is the erythematogenic effect of wavelength radiation (λ), I(λ) is the sun intensity at wavelength (λ), and is Abs(λ) the spectrophotometric reading of the absorbance of the sunscreen solution at the wavelength (λ).

#### 4.3.13 Evaluation of Cell Viability on L929 Fibroblast

NCTC clone 929 [L CELL, L‐929] murine fibroblast derived from normal subcutaneous areolar and adipose tissue) cells were obtained from Rio de Janeiro bank cells (BCRF‐0188) and maintained in DMEM supplemented with 10% FBS, 100 µg L^−1^ streptomycin, and 100 IU mL^−1^ penicillin, at 37°C in a 5% CO_2_. For cell viability, L929 fibroblast cells were plated at a density of 1 × 10^4^ cells per well and incubated at 37°C with 5% CO_2_ for 24 h to complete confluence. After, cells were exposed to CHE of *M. neoobscura* (1, 10, and 100 µg mL^−1^) or DMSO (10%) as positive control, and incubated again under same conditions. After incubation of 21 h, the cell viability was evaluated by MTT assay [74]. The percentage of viable cell was calculated and compared with negative control. The results were expressed as the percentage of cell viability relative to control.

#### 4.3.14 Evaluation of In Vitro Wound‐Healing Activity by Scratch Test

Murine L929 cells were seeded into each well of a 24‐well plate and incubated at 37°C with 5% CO_2_. After cell growth at high confluence in monolayer kept in the culture bottle, the cells will be trypsinized and seeded in 24‐well plates. After cell confluence, the culture medium was removed, and a continuous scratch was made in the medial surface of each well with a 200 µL tip. Then, the wells were washed with PBS to remove cell debris. The remaining cells were incubated with DMEM containing CHE of *M. neoobscura* (1, 10, or 100 µg mL^−1^). The analysis was performed by measuring the scratched area in mm^2^ at 0 time (immediately after mechanical trauma) and 24 h after incubation with the extract, by microscopy (Olympus CKX 41), using ImageJ 1.46r software. Analysis of the results will be carried out by calculating the % migration coverage area, using the formula:

$$\%\mathrm{of}24-h\mathrm{coverage}\mathrm{area}=\left(\mathrm{At}=0h\;-\;\mathrm{At}=\Delta h\right)\;\times \;100/\mathrm{At}\;=0h$$

where At = 0*h* is measured area immediately after scratching and At = Δ*h* is measured area 24 h after scratching. The results are expressed as the percent of the coverage area at 24 h in relation to time 0 h [75].

### 4.4 Evaluation of Toxicity of Samples

#### 4.4.1 HET‐CAM Assay

The HET‐CAM assay was performed according to ICCVAM [76]. Solution of 0.9% NaCl (w/v) as used as negative control and 1 M NaOH was used as positive control, DMSO (vehicle) in deionized water was used as a blank solution. CHE and fractions were tested at 300 µg mL^−1^ (dilute in DMSO). Fresh fertile clean eggs were candled before use and placed in an egg incubator (38°C and 60% relative humidity) for 8 days. After, air cell of the eggs were marked and cut off. CAM surfaces were directly exposed to test and an aliquot of 0.3 mL was deposited on the surface with the aid of a pipette. The hemorrhage, coagulation and vascular lysis were observed, photographed and recorded for 300 s (5 min). The numerical time‐individual scores for lysis, hemorrhage, and coagulation (Table 6) are summed to give a single numerical value indicating the irritation potential of the test substance on a scale with a maximum value of 21, where 0–0.9 is nonirritating, 1–4.9 is light irritating, 5–8.9 is moderate irritating, and 9–21 is several irritating. The assay was performed in triplicate.

**TABLE 6:** Individual score—HET‐CAM assay.

	Time		
	30 s	120 s	300 s
Damage	Score		
Vascular lysis	5	3	1
Hemorrhage	7	5	3
Coagulation	9	7	5

### 4.5 Irritation Assay by Agarose‐Overlay Method

The murine fibroblast cell line L929 (3 × 10^5^ cells/mL) was seeded in six‐well plates and incubated at 37°C in a humidified atmosphere with 5% CO_2_ until reaching confluence. Upon monolayer formation, the culture medium was removed and replaced with DMEM containing 0.01% neutral red as a vital dye, followed by 2‐h incubation in the dark. After dye removal and PBS washing, 3 mL of an overlay mixture (agarose:DMEM, 1:1.2) was added per well and allowed to solidify for 30 min. Filter‐sterilized paper discs (0.54 cm) impregnated with *M. neoobscura* extracts (1, 10, and 100 µg/mL) were placed at the center of each well. SDS and PBS served as positive and negative controls, respectively. Plates were incubated for 24 h at 37°C with 5% CO_2_. Irritation was assessed by measuring the lysis zone (absence of dye uptake) using a caliper, following the classification criteria established by the United States Pharmacopeia [77].

### 4.6 Statistical Analysis

For evaluation of enzyme‐inhibiting activity, the results were presented as the mean ± SD of three measurements. IC_50_ values were calculated using regression analysis as the concentrations of the samples that inhibited 50% of the respective enzyme activity and expressed in µg mL^−1^. The data obtained were analyzed using GraphPad Prism version 8.0 and expressed as mean ± SD. Statistical comparisons were made using one‐way ANOVA (GraphPad Prism), followed by Tukey's posttest, for the comparisons between the fractions, and Dunnett's posttest for the comparison with the control. *p* < 0.05 were considered statistically significant.

## Author Contributions

The study was conceptualized by **Michele Debiasi Alberton**. The methodology was designed by **Diogo Alexandre Siebert**, **André Luís de Gasper**, **José Roberto Santin**, **Isabel Daufenback Machado**, **Luciano Vitali** and **Marijana Zovko Končić**. The investigation was carried out by **Larissa Mascarenhas Krepsky**, **Larissa Benvenutti**, **Ana Helena Loos Moritz**, **Mayra Alice Corrêa Pitz**, **Letícia Bachmann**, **Paula Flegar**, **Stjepan Lešnjak**, **Ana Tirić**, **Amanda Tavares Germano**, **Mayara da Silva**, and **Carlos Rafael Vaz** Data curation was performed by **José Roberto Santin**, **Diogo Alexandre Siebert**, **Isabel Daufenback Machado**, **Luciano Vitali**, **Marijana Zovko Končić** and **Michele Debiasi Alberton**. The original draft was written by **Larissa Mascarenhas Krepsky**, **Marijana Zovko Končić**, and **Michele Debiasi Alberton** and the manuscript was reviewed and edited by **Michele Debiasi Alberton**. All authors have read and agreed to the published version of the manuscript.

## Conflicts of Interest

The authors declare no conflicts of interest.

## Data Availability

The data that support the findings of this study are available from the corresponding author upon reasonable request.
